# Adjuvant Therapy for Elderly Breast Cancer Patients after Breast-Conserving Surgery: Outcomes in Real World Practice

**DOI:** 10.3390/cancers15082334

**Published:** 2023-04-17

**Authors:** Paul Rogowski, Stephan Schönecker, Dinah Konnerth, Annemarie Schäfer, Montserrat Pazos, Aurélie Gaasch, Maximilian Niyazi, Edwin Boelke, Christiane Matuschek, Jan Haussmann, Michael Braun, Martin Pölcher, Rachel Würstlein, Nadia Harbeck, Claus Belka, Stefanie Corradini

**Affiliations:** 1 Department of Radiation Oncology, University Hospital LMU, 81377 Munich, Germany; 2 German Cancer Consortium (DKTK), 81377 Munich, Germany; 3 Department of Radiation Oncology, Heinrich Heine University, 40225 Dusseldorf, Germany; 4 Breast Centre, Red Cross Hospital, 80634 Munich, Germany; 5 Breast Centre, Department of Obstetrics and Gynecology, CCC Munich LMU, University Hospital LMU, 81377 Munich, Germany

**Keywords:** breast cancer, breast-conserving surgery, radiotherapy, endocrine therapy, local control, local recurrence, survival, outcome, elderly

## Abstract

**Simple Summary:**

The treatment of elderly patients with breast cancer often deviates from guideline recommendations due to comorbidities, expected side effects, and patient preference. We investigated the standard of care of postoperative radiotherapy after breast-conserving surgery in elderly patients (≥65 years) treated outside of clinical trials, potential factors related to the omission of radiotherapy, and the interaction with endocrine therapy. Overall, three thousand one hundred seventy-one women treated at two major breast centers were evaluated. Postoperative radiotherapy was performed in 82% of these cases. The irradiated patients were younger and more likely to receive additional endocrine therapy and chemotherapy. Patients who did not receive radiotherapy were significantly more likely to have non-invasive DCIS tumors and did not undergo axillary surgery. Radiotherapy was associated with improved locoregional tumor control, even in patients receiving endocrine therapy. Patients treated with radiotherapy alone had significantly better locoregional control than with endocrine therapy alone. In conclusion, the present work confirms the efficacy of postoperative radiotherapy in the elderly, even in patients receiving endocrine therapy.

**Abstract:**

We aimed to evaluate the standard of care of adjuvant radiotherapy (RT) after breast-conserving surgery (BCS) in elderly female patients (≥65 years) treated outside of clinical trials and to identify potential factors related to the omission of RT and the interaction with endocrine therapy (ET). All women treated with BCS at two major breast centers between 1998 and 2014 were evaluated. Data were provided by the Tumor Registry Munich. Survival analyses were conducted using the Kaplan–Meier method. Prognostic factors were identified using multivariate Cox regression analysis. The median follow-up was 88.4 months. Adjuvant RT was performed in 82% (2599/3171) of patients. Irradiated patients were younger (70.9 vs. 76.5 years, *p* < 0.001) and were more likely to receive additional chemotherapy (*p* < 0.001) and ET (*p* = 0.014). Non-irradiated patients more often had non-invasive DCIS tumors (pTis: 20.3% vs. 6.8%, *p* < 0.001) and did not undergo axillary surgery (no axillary surgery: 50.5% vs. 9.5%, *p* < 0.001). Adjuvant RT was associated with improved locoregional tumor control after BCS in invasive tumors (10-year local recurrence-free survival (LRFS): 94.0% vs. 75.1%, *p* < 0.001, 10-year lymph node recurrence-free survival (LNRFS): 98.1% vs. 93.1%, *p* < 0.001). Multivariate analysis confirmed significant benefits for local control with postoperative RT. Furthermore, RT led to increased locoregional control even in patients who received ET (10-year LRFS 94.8% with ET + RT vs. 78.1% with ET alone, *p* < 0.001 and 10-year LNRFS: 98.2% vs. 95.0%, *p* = 0.003). Similarly, RT alone had significantly better locoregional control rates compared to ET alone (10-year LRFS 92.6% with RT alone vs. 78.1% with ET alone, *p* < 0.001 and 10-year LNRFS: 98.0% vs. 95.0%, *p* = 0.014). The present work confirms the efficacy of postoperative RT for breast carcinoma in elderly patients (≥65 years) treated in a modern clinical setting outside of clinical trials, even in patients who receive ET.

## 1. Introduction

Radiation therapy (RT) is an important component of the multidisciplinary treatment of breast cancer and has been shown to improve locoregional tumor control and long-term survival in a number of randomised trials [1]. Although randomised trials set the gold standard for breast cancer treatments, elderly patients aged 65 years or older are substantially underrepresented in many clinical trials [2,3]. The omission of RT after breast-conserving surgery (BCS) in elderly patients with low-risk tumors receiving adjuvant endocrine therapy (ET) has been investigated in a series of randomised controlled trials [4,5,6,7,8]. However, randomised data comparing endocrine therapy alone vs. RT alone for older women with low-risk EBC are still sparse [9,10,11], although two trials are currently recruiting (EUROPA, NCT04134598 and CAMERAN, NCT054727929). Nevertheless, elderly patients who are included in clinical cancer trials tend to have fewer functional impairments or comorbidities than the average elderly patient treated in clinical practice [12].

For these reasons, the treatment of elderly patients with breast cancer in clinical practice often deviates from interdisciplinary therapy recommendations. In this context, treatment decisions are not exclusively influenced by tumor-specific parameters, but also by other factors such as comorbidities, expected toxicity, and patient preference. Frequently, physicians can choose from a range of available treatment options to select an appropriate treatment strategy for each individual patient. This results in a large heterogeneity of criteria influencing treatment decision-making in real-world settings. The aim of the present study was to evaluate treatment patterns and oncologic outcomes of postoperative RT after BCS in elderly female patients (≥65 years) treated outside of clinical trials. It also aimed to identify potential factors related to the omission of postoperative RT in daily clinical practice and the interaction with ET.

## 2. Materials and Methods

### 2.1. Data Sources

Data were obtained from the Munich Cancer Registry (MCR) [13]. The MCR systematically retrieved cancer reports from all MCR-affiliated hospitals or other reporting institutions. Patient demographics, cancer diagnosis, disease and treatment characteristics, and follow-up data were collected according to the official documentation guidelines for cancer registries. In addition, survival data were systematically retrieved from the death certificates of 23 local health authorities in the MCR catchment area. The catchment area of the population-based clinical cancer registry included nearly 5 million inhabitants.

### 2.2. Study Population

For the present study, all breast cancer patients with an age of ≥65 years and treated with BCS between 1 January 1998 and 31 December 2014 at two breast cancer centres in Southern Germany were analysed. Male patients in cases with histology of lymphoma, sarcoma, unknown date of initial diagnosis (e.g., tumors from death certificate information only), a previous diagnosis of cancer, and concurrent cancer diagnoses or distant metastases were excluded from the present analysis. The final study cohort consisted of 3171 women.

### 2.3. Statistical Analyses and Endpoints

Statistical analyses were conducted using IBM SPSS Statistics 25.0 (Armonk, New York, NY, USA). Oncological outcome analysis was performed for invasive tumors only. Descriptive statistics were used to analyse patient and treatment characteristics and data were compared using the chi-squared test. Primary endpoints were the impact of RT on locoregional control concerning local recurrence-free survival (LRFS) and lymph node recurrence-free survival (LNRFS). Distant metastasis-free survival (DMFS) and overall survival (OS) were also estimated using the Kaplan–Meier method and compared using the log-rank test. The observation period began after the diagnosis of the invasive tumor and ended at the date of LR/LNR/distant metastasis occurrence, date of death, or the last follow-up for cases without events. Multivariate Cox proportional hazards models were used to account for competing risks in order to identify independent prognostic factors related to LRFS, LNRFS, DMFS, and OS. Furthermore, factors influencing the use of postoperative RT were determined by using a multivariate logistic regression analysis. The significance level in all analyses was set at 5%.

## 3. Results

### 3.1. Patient Characteristics

An overview of patient characteristics is presented in Table 1. Adjuvant RT was performed in 82% (2599/3171) of patients following BCS. While the tumor side was balanced between the groups (right-sided 47% vs. left-sided 53%, *p* = 0.799), there were a variety of significant differences between patients undergoing surgery without RT and patients receiving postoperative RT (see Table 1).

Patients undergoing BCS with adjuvant RT were significantly younger (median 70.9 years vs. 76.5 years, *p* < 0.001). Moreover, patients treated with BCS without RT were significantly more likely to have ductal carcinoma in situ (DCIS, 20.3% vs. 6.8%) and did not undergo axillary surgery (50.5% vs. 9.5%). In contrast, patients treated with BCS and RT typically received sentinel node biopsy (43.8%) and were pN0 (60.9%) at pathological examination. Regarding other treatment modalities, irradiated patients received ET in 52.3% of cases. In contrast, in patients not undergoing RT, adjuvant ET was administered only in 37.4% (*p* < 0.001) of cases, although there was no significant difference in the presence of hormone receptor positivity (89.3% vs. 90.3%, *p* = 0.505). Chemotherapy was also administered more frequently in patients undergoing RT (19% vs. 6.6%, *p* < 0.001).

Furthermore, treatment was age-dependent in patients with invasive tumors (see Table 2). Older patients were less likely to receive axillary surgery, RT, and chemotherapy. In contrast, there was no difference between age groups regarding the administration of ET. Axillary surgery was carried out in 98%, 94.7%, and 71.8% of patients aged <70 years, 70–75 years, and >75 years, respectively (*p* < 0.001). RT was used in 92.8%, 91.3%, and 62.5% of women in the respective age groups (*p* < 0.001). The same tendency was seen with 23.4%, 12.9%, and 4.2% of patients in those age groups receiving chemotherapy (*p* < 0.001). The comparison of combined adjuvant therapies for different age groups also showed a significant change from adjuvant RT, to adjuvant ET alone, or to no adjuvant therapy at all with increasing age.

### 3.2. Outcome

The median follow-up was 88.4 months. An overview of the Kaplan–Meier estimates is given in Table 3, and multivariate Cox regression analysis is in Table 4.

**Figure 1:**
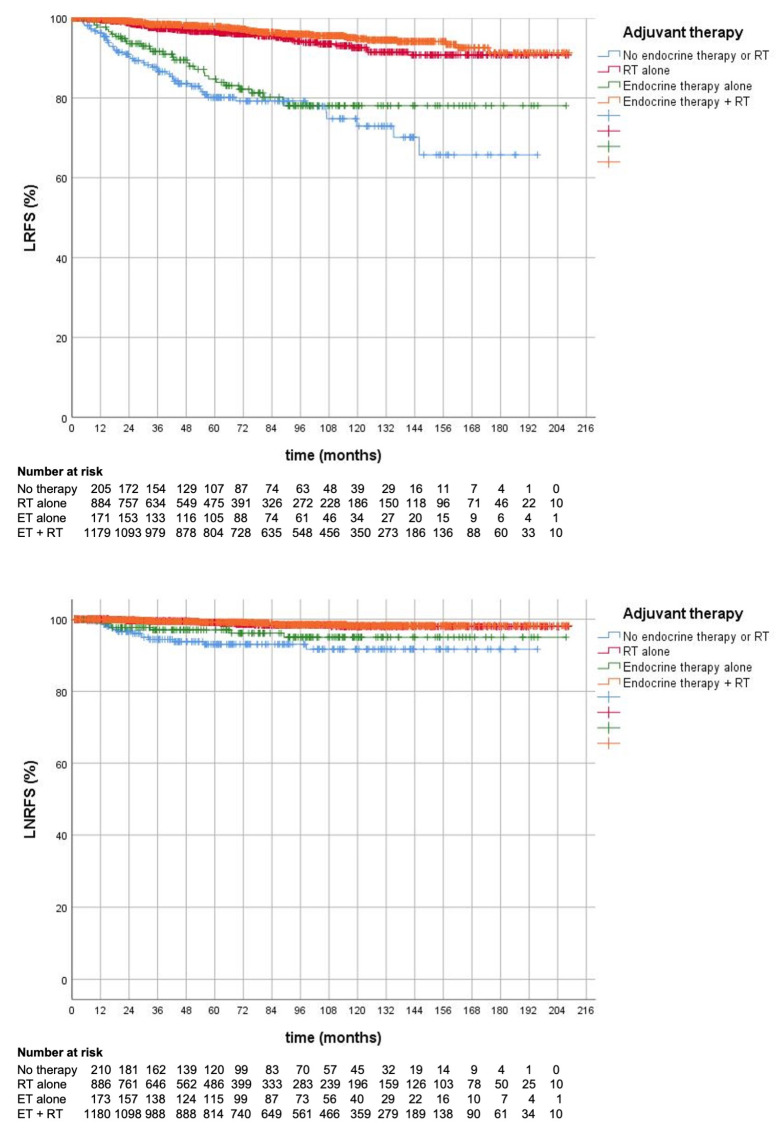
Kaplan–Meier analysis for loco-regional recurrence-free survival (LRFS) and lymph node recurrence-free survival (LNRFS) for invasive carcinomas who underwent breast-conserving surgery (*n* = 2684).

Overall, patients with invasive tumors (*n* = 2684) receiving adjuvant RT showed longer local recurrence and lymph node recurrence-free survival rates after BCS on univariate analysis (10-year LRFS: 94.0% BCS + RT vs. 75.1% BCS without RT, *p* < 0.001, 10-year LNRFS: 98.1% BCS + RT vs. 93.1% BCS without RT, *p* <0.001, see Table 3 and Table 4 and Figure 1), which was also confirmed by the multivariate analysis, where adjuvant RT was associated with improved LRFS (hazard ratio [HR] 0.180; 95% confidence interval [CI], 0.119–0.275, *p* < 0.001) and LNRFS (HR 0.306; 95%CI: 0.129–0.729, *p* = 0.008). A subgroup analysis showed that this was significant for all analysed age groups (<70 years: 10-year LRFS 95.5% vs. 79.2%, 70–74 years: 92.5% vs. 67.0%, ≥75 years: 90.3% vs. 76.2%, *p* < 0.001 respectively for all subgroups).

The effect on local control was still present in patients with invasive tumors who received ET; they still had a significantly increased LRFS after postoperative RT (10-year LRFS 94.8% with ET + RT vs. 78.1% with ET alone, *p* < 0.001). Similarly, patients receiving ET and undergoing RT showed longer LNRFS compared to surgery and ET alone (10-year LNRFS 98.2% with ET + RT vs. 95.0% ET alone, *p* = 0.003). Moreover, RT alone also had significantly better locoregional control rates compared to ET alone (10-year LRFS 92.6% with RT alone vs. 78.1% with ET alone, *p* < 0.001 and 10-year LNRFS: 98.0% vs. 95.0%, *p* = 0.014). Table 4 summarizes other classic prognostic risk factors that were associated with prolonged LNRFS on multivariate analysis, such as a small tumor stage (*p* < 0.001) and positive hormone receptor status (*p* = 0.009).

Ten-year DMFS for patients with invasive tumors was 89.0% in the BCS + RT group, compared to 76.6% in the BCS without RT group (*p* < 0.001). Other factors related to poor DMFS on multivariate Cox regression analysis were an advanced tumor stage (*p* < 0.001), positive nodal status (*p* < 0.001), high tumor grade (*p* < 0.001), and older age (*p* = 0.012, Table 4).

Patients receiving postoperative RT following BCS had a longer 10-year OS (76.4%) compared to patients undergoing breast-conserving surgery without RT (39.3%) (*p* < 0.001). After adjusting for risk factors in the Cox proportional hazards regression model, postoperative RT (HR 0.613; 95%CI: 0.469–0.801, *p* < 0.001) and administration of ET (*p* = 0.010) were correlated with longer OS. In contrast, tumor-specific risk factors like advanced tumor stage (*p* < 0.001), positive nodal status (*p* < 0.001), and high tumor grade (*p* = 0.010), as well as advanced age (*p* < 0.001), were correlated with shorter OS (Table 4).

### 3.3. Factors Associated with the Use of Postoperative Radiotherapy

A multivariate logistic regression analysis was performed to identify factors associated with the administration of postoperative RT (Table 5). Advanced age at diagnosis (≥75 years, OR: 0.310, 95%CI: 0.215–0.446) and advanced positive nodal status (pN+ with ≥4 positive lymph nodes, OR 0.353, 95% CI: 0.198–0.630) were associated with a nearly 70% reduction in the odds of receiving postoperative RT following BCS. Furthermore, administration of other therapies such as chemotherapy and/or ET was associated with a two-fold higher likelihood of also receiving postoperative RT.

## 4. Discussion

In the absence of randomized evidence for elderly patients, clinicians have to choose from a range of possible treatment strategies usually extrapolated from clinical trials conducted in younger, healthier patients. Furthermore, shared decision-making in daily clinical practice is strongly influenced by a number of unpredictable confounding factors, such as clinician and patient preferences and trade-offs concerning toxicity risks or comorbidities, as well as the subjective burden of planned cancer treatments [14,15].

In the setting of breast-conserving surgery, the EBCTCG meta-analysis showed that postoperative RT halves the 10-year rate of any breast cancer recurrence (35% vs. 19%) and reduces the 15-year breast cancer-related mortality by about a sixth (25% vs. 21%) [1]. This was seen for all subgroups; however, the proportional absolute benefit for elderly patients was smaller. Women aged 60–69 years had an absolute gain through postoperative RT in a 10-year rate of any recurrence of 14.1 % (*p* < 0.001), while in patients ≥70 years the absolute gain was only 8.9 % (*p* < 0.001). This is in line with the results of the current study, in which elderly patients ≥65 years had better local tumor control and fewer lymph node recurrences when receiving adjuvant RT (LRFS HR: 0.180; 95%CI: 0.119–0.275, *p* < 0.001 and LNRFS HR: 0.306; 95%CI: 0.129–0.729, *p* = 0.008). A subgroup analysis showed that this was significant for all analysed age groups (<70 years; 70–74 years and ≥75 years).

The observation that elderly patients benefit less from RT led to a series of randomized controlled trials [4,5,6,7,8] investigating the omission of RT in elderly patients receiving adjuvant ET. This was limited to tumors with favourable prognostic factors (“low-risk”, mostly T1 tumors, node-negative, hormone-receptor-positive, low grade, age ≥ 65–70 years). Most of these studies showed a significant increase in local tumor recurrence rates when RT was omitted, even if overall survival was not compromised. A meta-analysis of five of those randomized controlled trials with a total of three thousand seven hundred sixty-six patients found a seven-fold increase in the risk of local recurrence in patients treated with ET alone [16]. However, a frequent problem encountered in clinical practice is early discontinuation and suboptimal adherence to ET. In several studies, it was observed that only 50–65% of patients with breast cancer maintained ET within the first 5 years [17,18,19,20,21]. Older age and comorbidities were associated with earlier discontinuation [17,18,19]. An analysis of 961 patients aged ≥65 years found that 49% of them discontinued ET. Patients who did not adhere to ET were more likely to be 75–80 years (HR 1.41, 95%CI: 1.06–1.87), over 80 years old (HR 2.02; 95%CI: 1.53–2.66), or have an increase in their Charlson Comorbidity Index at 3 years from diagnosis (HR 1.52; 95%CI: 1.18–1.95) [19]. Therefore, the results from clinical trials regarding the omission of RT may not be directly applicable to clinical practice due to the poor adherence rates to ET in elderly patients.

This can also be observed in our real-world cohort. Overall, omission of RT after BCS was more common in elderly patients older than 75 years (38%) compared to 7% in patients aged <70 years or 9% among 70–75-year-old patients, confirming a wide variation in clinical practice patterns across different age cohorts. The same trend was observed for the omission of axillary surgery and chemotherapy. Regarding ET, overall, about 58% of patients with hormone-receptor-positive invasive tumors received ET, with an equal distribution across all age groups. However, the proportion of patients who did not undergo ET or adjuvant RT after BCS was higher in patients >75 years (18%) compared to only 5% in patients aged 65–69 years. This was also seen in the logistic regression analysis, where advanced age at diagnosis (≥75 years) was associated with a nearly 70% reduced likelihood of receiving postoperative RT following BCS (OR: 0.310, 95%CI: 0.215–0.446). When comparing local control outcomes regarding the application of ET and RT, RT was also correlated with prolonged LRFS in patients receiving ET (10-year LRFS 94.3% with RT vs. 76.9% without RT, *p* < 0.001). Similarly, these patients also had longer locoregional lymph node recurrence-free survival rates compared to surgery and ET alone (10-year LNRFS 98.2% with RT vs. 95.5% without RT, *p* = 0.011). Figure 1 shows that local control in patients with hormone-receptor-positive tumors undergoing ET was comparable to the group of patients not receiving any adjuvant treatment (*p* = 0.240). In contrast, the addition of RT was associated with significantly improved LRFS, irrespective of the addition of ET (*p* < 0.001). Taken together, we could show in this clinical cohort that RT after BCS in invasive carcinomas significantly influences local tumor control, even if adjuvant ET is administered. As the omission of RT is associated with a significant increase in local tumor recurrence rates, omission of RT in elderly patients with early breast cancer should mainly be restricted to frail patients with a reduced life expectancy (<10 years). For all other elderly patients, undertreatment should be avoided. Whether ET can be de-escalated in contrast to the omission of RT still needs further clinical investigation. Unfortunately, in our data set, we could not ascertain all reasons which may have influenced the clinical treatment decision-making process such as host-related factors including comorbidities, poor performance status, or clinician- and patient-related preferences.

The findings have to be interpreted with caution, as the retrospective nature of this study has inherent limitations: clinical-effectiveness study outcomes are influenced by a variety of unmeasured confounding factors, and associations between treatments and outcomes might arise from these and they have no causal correlation [22,23,24,25]. We attempted to account for all known confounding factors available in the registry, which may have influenced the clinical treatment decision-making. However, host-related factors cannot be completely accounted for. Therefore, an unavoidable limitation is a potential selection bias due to disease severity [26] considering that, for example, the presence of high-risk factors is a potential confounder impacting the indication for postoperative RT. However, only patients with invasive carcinoma were considered for the oncological outcome evaluation. Nevertheless, there were significantly more patients in the postoperative radiation group of the current study with high-risk characteristics such as higher tumor size or known positive nodal status. We tried to account for these clinicopathological risk-score characteristics in multivariate analysis. However, since molecular subtypes and histopathological parameters were not available for all patients from the registry, an accurate risk-group stratification to define baseline risks for local recurrence was not possible. Furthermore, data on adherence and treatment duration for patients treated with ET were not available. Lastly, systemic therapy in early breast cancer has been improving substantially over the last few years so that outcomes may look different in more recently treated populations.

## 5. Conclusions

The present work confirms the efficacy of postoperative RT for breast carcinoma in elderly patients (≥65 years) treated in a modern clinical setting outside of clinical trials, even in patients who receive ET.

## Figures and Tables

**Table 1:** Patient and tumor characteristics for the entire cohort. BCS: breast-conserving surgery; RT: radiation therapy; ALND: axillary lymph node dissection; SNB: sentinel node biopsy.

	BCS Alone		BCS + RT		*p*-Value
	*n*	(%)	*n*	(%)	
All	572	(18.0)	2599	(82.0)	
Age at diagnosis					<0.001
65–69 years	141	(24.7)	1267	(48.7)	
70–75 years	122	(21.3)	523	(32.8)	
>75 years	309	(54.0)	479	(18.4)	
median (years)	76.5		70.9		
Lateralisation					0.799
right	268	(46.9)	1233	(47.4)	
left	304	(53.1)	1366	(52.6)	
Tumor size					<0.001
pTis	116	(20.3)	176	(6.8)	
pT1	239	(41.8)	1534	(59.0)	
pT2	170	(29.7)	697	(26.8)	
pT3	3	(0.5)	24	(0.9)	
pT4	10	(1.7)	7	(0.3)	
unknown/pTx	34	(5.9)	161	(6.2)	
Nodal status					<0.001
pN0	178	(31.1)	1584	(60.9)	
pN1	64	(11.2)	506	(19.5)	
pN2	17	(3.0)	83	(3.2)	
pN3	6	(1.0)	35	(1.3)	
unknow/pNx	307	(53.7)	391	(15.0)	
Grade					0.709
G1	77	(16.8)	432	(17.7)	
G2	276	(60.4)	1492	(61.2)	
G3	104	(22.8)	515	(21.1)	
Axillary surgery					<0.001
None	289	(50.5)	246	(9.5)	
ALND	111	(19.4)	696	(26.8)	
SNB + ALND	49	(8.6)	408	(15.7)	
SNB alone	105	(18.4)	1139	(43.8)	
other	18	(3.1)	110	(4.2)	
Resection status					<0.001
R0	425	(70.7)	2173	(83.6)	
R1/R2	22	(3.1)	57	(2.2)	
* *Rx	125	(26.2)	369	(14.2)	
Hormone receptor					0.505
positive	452	(89.3)	2298	(90.3)	
negative	54	(10.7)	247	(9.7)	
Chemotherapy					<0.001
no	534	(93.4)	2105	(81.0)	
yes	38	(6.6)	494	(19.0)	
Endocrine therapy					<0.001
no	358	(62.6)	1240	(47.7)	
yes	214	(37.4)	1359	(52.3)	

**Table 2:** Treatment characteristics divided per age group for patients with invasive tumors (*n* = 2684). All patients underwent breast-conserving surgery.

	<70 Years		70–75 Years		>75 Years		*p*-Value
	*n*	(%)	*n*	(%)	*n*	(%)	
All	1155	(43.0)	815	(30.4)	714	(26.6)	
Axillary surgery							<0.001
yes	1132	(98.0)	772	(94.7)	513	(71.8)	
no	23	(2.0)	43	(5.3)	201	(28.2)	
Radiation Therapy							<0.001
yes	1072	(92.8)	744	(91.3)	446	(62.5)	
none	83	(7.2)	71	(8.7)	268	(37.5)	
Chemotherapy							<0.001
yes	270	(23.4)	105	(12.9)	30	(4.2)	
no	885	(76.6)	710	(87.1)	684	(95.8)	
Endocrine therapy							0.238
yes	617	(53.4)	456	(56.0)	369	(51.7)	
no	538	(46.6)	359	(44.0)	345	(48.3)	
Adjuvant therapy							<0.001
ET alone	26	(2.3)	28	(3.4)	138	(19.3)	
ET + RT	591	(51.2)	428	(52.5)	231	(32.4)	
RT alone	481	(41.6)	316	(38.8)	215	(30.1)	
No adj. therapy	57	(4.9)	43	(5.3)	130	(18.2)	

**Table 3:** Kaplan–Meier estimates of local recurrence-free survival (LRFS), lymph node recurrence-free survival (LNRFS), distant metastasis-free survival (DMFS), and overall survival (OS) for patients with invasive tumors (*n* = 2684) following breast-conserving surgery (BCS). y: years; RT: radiation therapy; ET: endocrine therapy; adj. therapy: adjuvant therapy.

		5 y (%)	10 y (%)	*p*-Value
LRFS				
RT	BCS + RT	97.3	94.0	<0.001
	BCS alone	81.8	75.1	
Adjuvant Therapy	ET alone	84.7	78.1	
	ET + RT	97.9	94.8	<0.001
	RT alone	96.5	92.6	<0.001
	No adj. therapy	80.1	72.9	0.240
LNRFS				
RT	BCS + RT	99.2	98.1	<0.001
	BCS alone	94.9	93.1	
Adjuvant Therapy	ET alone	97.0	95.0	
	ET + RT	99.2	98.2	0.003
	RT alone	99.1	98.0	0.014
	No adj. therapy	93.0	91.6	0.215
DMFS				
	BCS + RT	93.5	89.0	<0.001
	BCS alone	84.8	76.6	
Adjuvant Therapy	ET alone	81.8	73.1	
	ET + RT	93.0	88.3	<0.001
	RT alone	94.4	90.2	<0.001
	No adj. therapy	87.4	79.8	0.130
OS				
	BCS + RT	91.7	76.4	<0.001
	BCS alone	71.2	39.3	
Adjuvant Therapy	ET alone	69.3	36.0	
	ET + RT	92.0	76.8	<0.001
	RT alone	91.4	75.0	<0.001
	No adj. therapy	72.8	41.7	0.383

**Table 4:** Multivariate Cox regression analysis for loco-regional recurrence-free survival (LRFS), lymph node recurrence-free survival (LNRFS), distant metastasis-free survival (DMRFS), and overall survival (OS) for patients with invasive carcinomas who underwent breast-conserving surgery (*n* = 2684).

		LRFS			LNRFS			DMRFS			OS	
Variable	HR	95% CI	*p*	HR	95% CI	*p*	HR	95% CI	*p*	HR	95% CI	*p*
Age			0.507			0.156			0.012			<0.001
<70 years	1			1			1			1		
70–74 years	1.135	0.706–1.826		2.462	0.985–6.154		1.213	0.841–1.750		1.726	1.339–2.224	
≥75 years	1.338	0.820–2.183		1.717	0.608–4.853		1.824	1.226–2.715		3.513	2.702–4.568	
Tumor size			0.097			<0.001			<0.001			<0.001
pT1	1			1			1			1		
pT2	1.537	1.022–2.311		2.476	1.052–5.830		2.389	1.700–3.357		1.534	1.234–1.906	
pT3–4	0.700	0.093–5.277		15.355	4.059–58.081		4.310	2.123–8.748		1.933	0.96–3.878	
Nodal status			0.156			0.132			<0.001			<0.001
pN0	1			1			1			1		
pN+ (<3 LN)	1.504	0.976–2.318		2.320	1.022–5.264		2.295	1.621–3.250		1.608	1.280–2.019	
pN+ (≥4 LN)	0.938	0.392–2.245		1.677	0.414–6.792		3.788	2.383–6.021		1.898	1.280–2.813	
Grade			0.219			0.474			<0.001			0.001
1	1			1			1			1		
2	1.499	0.796–2.821		0.838	0.229–3.070		1.376	0.759–2.494		1.195	0.877–1.628	
3	1.934	0.920–4.065		1.436	0.347–6.175		3.116	1.666–5.830		1.898	1.266–2.603	
Resection status			0.478			0.408			0.455			0.331
R0	1			1			1			1		
R1/2	0.656	0.205–2.102		0.399	0.045–3.519		1.288	0.662–2.506		1.271	0.784–2.063	
Hormone receptor status			0.123			0.009			0.338			0.755
negative	1			1			1			1		
positive	0.601	0.315–1.149		0.214	0.068–0.676		0.766	0.444–1.321		1.063	0.726–1.556	
Radiotherapy			<0.001			0.008			0.042			<0.001
no	1			1			1			1		
yes	0.180	0.119–0.275		0.306	0.129–0.729		0.655	0.436–0.984		0.613	0.469–0.801	
Chemotherapy			0.091			0.102			0.627			0.611
no	1			1			1			1		
yes	0.591	0.322–1.088		0.380	0.119–1.213		0.907	0.612–1.344		0.926	0.689–1.245	
Endocrine Therapy			0.125			0.819			0.240			0.010
no	1			1			1			1		
yes	0.710	0.459–1.099		0.892	0.336–2.366		1.250	0.862–1.815		0.735	0.583–0.928	

**Table 5:** Adjusted Odd Ratios (OR) and 95% Confidence Intervals (CI) from the multiple logistic regression analysis for the administration of postoperative radiotherapy.

		BCS	
Variable	OR	95% CI	*p*-Value
Age			<0.001
<70 years	1		
70–74 years	1.001	0.665–1.506	
≥75 years	0.310	0.215–0.446	
Tumor size			0.142
pT1	1		
pT2	0.713	0.509–0.997	
pT3-4	0.807	0.263–2.471	
Nodal status			0.002
pN0	1		
pN+ (<3 LN)	0.762	0.528–1.098	
pN+ (≥4 LN)	0.353	0.198–0.630	
Grade			0.274
1	1		
2	0.751	0.474–1.190	
3	0.628	0.355–1.110	
Resection status			0.230
R0	1		
R1/2	0.623	0.288–1.349	
Hormone receptor status			0.529
negative	1		
positive	1.196	0.685–2.087	
Chemotherapy			0.001
no	1		
yes	2.484	1.435–4.299	
Endocrine Therapy			<0.001
no	1		
yes	1.928	1.394–2.665	

## Data Availability

Data are contained within the article.

## References

[1] Darby S., McGale P., Correa C., Taylor C., Arriagada R., Clarke M., Cutter D., Davies C., Ewertz M., Godwin J. (2011). Effect of radiotherapy after breast-conserving surgery on 10-year recurrence and 15-year breast cancer death: Meta-analysis of individual patient data for 10 801 women in 17 randomised trials. Lancet.

[2] Hutchins L.F., Unger J.M., Crowley J.J., Coltman C.A.J., Albain K.S. (2008). Underrepresentation of Patients 65 Years of Age or Older in Cancer-Treatment Trials. N. Engl. J. Med..

[3] Kemeny M.M., Peterson B.L., Kornblith A.B., Muss H.B., Wheeler J., Levine E., Bartlett N., Fleming G., Cohen H.J. (2003). Barriers to clinical trial participation by older women with breast cancer. J. Clin. Oncol..

[4] Fyles A.W., McCready D.R., Manchul L.A., Trudeau M.E., Merante P., Pintilie M., Weir L.M., Olivotto I.A. (2004). Tamoxifen with or without Breast Irradiation in Women 50 Years of Age or Older with Early Breast Cancer. N. Engl. J. Med..

[5] Blamey R.W., Bates T., Chetty U., Duffy S.W., Ellis I.O., George D., Mallon E., Mitchell M.J., Monypenny I., Morgan D.A.L. (2013). Radiotherapy or tamoxifen after conserving surgery for breast cancers of excellent prognosis: British Association of Surgical Oncology (BASO) II trial. Eur. J. Cancer.

[6] Hughes K.S., Schnaper L.A., Bellon J.R., Cirrincione C.T., Berry D.A., McCormick B., Muss H.B., Smith B.L., Hudis C.A., Winer E.P. (2013). Lumpectomy plus tamoxifen with or without irradiation in women age 70 years or older with early breast cancer: Long-term follow-up of CALGB 9343. J. Clin. Oncol..

[7] Fastner G., Sedlmayer F., Widder J., Metz M., Geinitz H., Kapp K., Fesl C., Sölkner L., Greil R., Jakesz R. (2020). Endocrine therapy with or without whole breast irradiation in low-risk breast cancer patients after breast-conserving surgery: 10-year results of the Austrian Breast and Colorectal Cancer Study Group 8A trial. Eur. J. Cancer.

[8] Kunkler I.H., Williams L.J., Jack W.J.L., Cameron D.A., Dixon J.M. (2023). Breast-Conserving Surgery with or without Irradiation in Early Breast Cancer. N. Engl. J. Med..

[9] Joseph K., Zebak S., Alba V., Mah K., Au C., Vos L., Ghosh S., Abraham A., Chafe S., Wiebe E. (2021). Adjuvant breast radiotherapy, endocrine therapy, or both after breast conserving surgery in older women with low-risk breast cancer: Results from a population-based study. Radiother. Oncol..

[10] Murphy C.T., Li T., Wang L.S., Obeid E.I., Bleicher R.J., Eastwick G., Johnson M.E., Hayes S.B., Weiss S.E., Anderson P.R. (2015). Comparison of Adjuvant Radiation Therapy Alone Versus Radiation Therapy and Endocrine Therapy in Elderly Women with Early-Stage, Hormone Receptor-Positive Breast Cancer Treated with Breast-Conserving Surgery. Clin. Breast Cancer.

[11] Fisher B., Bryant J., Dignam J.J., Wickerham D.L., Mamounas E.P., Fisher E.R., Margolese R.G., Nesbitt L., Paik S., Pisansky T.M. (2002). Tamoxifen, radiation therapy, or both for prevention of ipsilateral breast tumor recurrence after lumpectomy in women with invasive breast cancers of one centimeter or less. J. Clin. Oncol..

[12] Sedrak M.S., Freedman R.A., Cohen H.J., Muss H.B., Jatoi A., Klepin H.D., Wildes T.M., Le-Rademacher J.G., Kimmick G.G., Tew W.P. (2021). Older adult participation in cancer clinical trials: A systematic review of barriers and interventions. CA. Cancer J. Clin..

[13] MCR Munich Cancer Registry. https://www.tumorregister-muenchen.de/.

[14] Lyman G.H., Levine M. (2012). Comparative effectiveness research in oncology: An overview. J. Clin. Oncol..

[15] Armstrong K. (2012). Methods in comparative effectiveness research. J. Clin. Oncol..

[16] Matuschek C., Bölke E., Haussmann J., Mohrmann S., Nestle-Krämling C., Gerber P.A., Corradini S., Orth K., Kammers K., Budach W. (2017). The benefit of adjuvant radiotherapy after breast conserving surgery in older patients with low risk breast cancer- a meta-analysis of randomized trials. Radiat. Oncol..

[17] Nekhlyudov L., Li L., Ross-Degnan D., Wagner A.K. (2011). Five-year patterns of adjuvant hormonal therapy use, persistence, and adherence among insured women with early-stage breast cancer. Breast Cancer Res. Treat..

[18] Hershman D.L., Kushi L.H., Shao T., Buono D., Kershenbaum A., Tsai W.Y., Fehrenbacher L., Lin Gomez S., Miles S., Neugut A.I. (2010). Early discontinuation and nonadherence to adjuvant hormonal therapy in a cohort of 8,769 early-stage breast cancer patients. J. Clin. Oncol..

[19] Owusu C., Buist D.S.M., Field T.S., Lash T.L., Thwin S.S., Geiger A.M., Quinn V.P., Frost F., Prout M., Yood M.U. (2008). Predictors of tamoxifen discontinuation among older women with estrogen receptor-positive breast cancer. J. Clin. Oncol..

[20] Barron T.I., Connolly R.M., Bennett K., Feely J., Kennedy M.J. (2007). Early discontinuation of tamoxifen: A lesson for oncologists. Cancer.

[21] Partridge A.H., Wang P.S., Winer E.P., Avorn J. (2003). Nonadherence to adjuvant tamoxifen therapy in women with primary breast cancer. J. Clin. Oncol..

[22] Corradini S., Bauerfeind I., Belka C., Braun M., Combs S.E., Eckel R., Harbeck N., Hölzel D., Kiechle M., Niyazi M. (2017). Trends in use and outcome of postoperative radiotherapy following mastectomy: A population-based study. Radiother. Oncol..

[23] Corradini S., Niyazi M., Niemoeller O.M., Li M., Roeder F., Eckel R., Schubert-Fritschle G., Scheithauer H.R., Harbeck N., Engel J. (2015). Adjuvant radiotherapy after breast conserving surgery—A comparative effectiveness research study. Radiother. Oncol..

[24] McGale P., Cutter D., Darby S.C., Henson K.E., Jagsi R., Taylor C.W. (2016). Can observational data replace randomized trials?. J. Clin. Oncol..

[25] Giordano S.H., Kuo Y.F., Duan Z., Hortobagyi G.N., Freeman J., Goodwin J.S. (2008). Limits of observational data in determining outcomes from cancer therapy. Cancer.

[26] Salas M., Hofman A., Stricker B.H. (1999). Confounding by indication: An example of variation in the use of epidemiologic terminology. Am. J. Epidemiol..

